# A novel viroid-like RNA element “Obelisks”: a major breakthrough in the RNA World

**DOI:** 10.1186/s43556-025-00290-7

**Published:** 2025-07-04

**Authors:** Li Li, Xinqin Zhang, Min Wu

**Affiliations:** 1 Department of Respiratory Medicine, Daping Hospital, Third Military Medical University (Army Medical University), Chongqing, 400042 China; 2 Department of Trauma Medical Center, Daping Hospital, State Key Laboratory of Trauma and Chemical Poisoning, Army Medical University, Chongqing, 400042 China; 3 Wenzhou Institute, University of Chinese Academy of Sciences, Wenzhou, Zhejiang 325000 China

In a recent study published in *Cell*, Zheludev et al. [1] identified a novel class of RNA elements, termed "Obelisks", which function as autonomous replicons encoding self-cleaving ribozymes and a previously uncharacterized protein family-"Oblins". These rod-structured elements (< 1 kb) represent the smallest known autonomous RNA replicons discovered within animal ecosystems. Their discovery uncovers a hidden RNA biosphere, redefining minimal genomes and offering new insights into RNA evolution, as well as host-microbe interaction.

Previous studies have suggested that viroids are one of the smallest known entities which can perform many basic functions of life and may have played a role in evolution since the beginning of life on Earth [2]. However, the detection and analysis methods used in past studies mostly relied on known sequence databases for comparison and identification of new analogs. In the event that a new viroid or its variant is not similar to any sequence in the existing database, it is at high risk of being ignored, meaning that there may exist undiscovered viroids or other microbial entities.

Instead of relying on solely sequence-matching-based strategies, Zheludev et al. developed a specialized bioinformatic method, the Viroid Nominator (VNom), which is defined as apparent circularity, and the co-occurrence of both positive and negative-sense strands within a given sample. Based on its multi-level screening and verification process, coupled with automated and high-throughput processing capabilities, a new class of RNA elements—"Obelisks", has been discovered in the data of the Human Microbiome Project (iHMP) with higher sensitivity and comprehensiveness.

Previous research considered viroids to exist only in plants, although a recent study unveiled a novel viroid-like circular RNA in the tumor tissues of colorectal cancer patients, which is regarded as a mammalian viroid and named colorectal cancer-associated viroid (CCAV)[3]. The biological distribution of viroids has not been fully known yet, while Obelisks are widely present in a variety of ecosystems. Their presence in the human microbiome is a particularly interesting finding. In the human fecal and oral microbiome transcriptome data studied, Obelisks achieved detection rates of about 7% and 50%, respectively. Through further analysis, researchers determined that the oral microorganism *Streptococcus sanguinis* is the host of a specific Obelisk, suggesting the replication ability of Obelisks in a specific bacterium. As for its structural composition, the RNA genome of Obelisks is approximately 1 kb and has a circular and rod-shaped secondary structure. More importantly, these RNA molecules contain a new superfamily coding region of the protein, which the researchers named "Oblin". This characteristic is similar to Hepatitis Delta Virus, which also has a viroid-like genome structure with a coding region. In addition, some Obelisks also carry mutated hammerhead ribozyme sites, suggesting that they may have self-splicing capabilities, similar to the self-replication mechanisms of some virus-like organisms. Based on the characteristics of themselves - cleaving ribozymes and precise gene-cutting, recombination systems may be designed enabling the development of new genetic engineering tools.

Despite these promising findings, it is important to note that many questions arise about the Obelisks. Although Obelisks have been detected in multiple samples, their specific biological functions are not well understood. The oral bacterium Streptococcus sanguinis can be used to deeply study the functions of Obelisks, including how they affect host cell metabolism, immune responses, and what role their proteins play, etc. (Fig. 1). In addition, the evolutionary history and origin of Obelisks are still unclear, which limits the understanding of their biological significance. Through comparative genomics and phylogenetic analysis, their evolutionary relationships with known viruses and viroids may be identified by reconstructing the evolutionary tree of Obelisks. Furthermore, are there any other undiscovered members in its family? A recent study revealed the extensive diversity and ecological distribution of viroid-like circular RNA agents through metatranscriptome mining [4]. Another study increased the diversity of viroid cccRNAs by five-fold through a large-scale analysis of metatranscriptomic data. We might refer to this method to design analytical tools to further explore Obelisks’ diversity and ecological distribution [5].

**Fig. 1:**
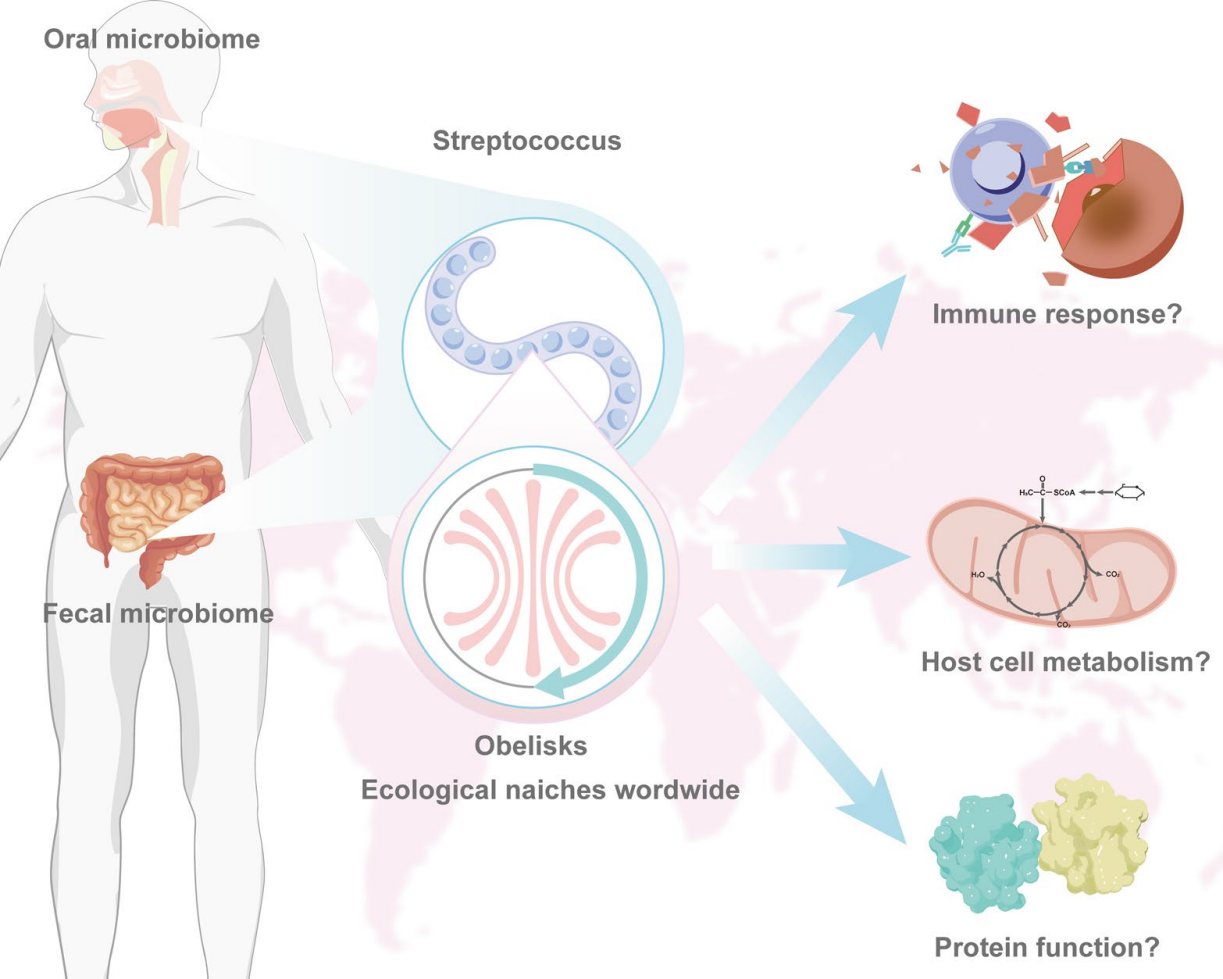
Potential biological functions of Obelisks. Obelisks exist in a variety of ecological niches around the world, including in the human fecal and oral microbiomes. As the host of a specific Obelisk, the oral bacterium *Streptococcus sanguinis* may be used to deeply study the functions of Obelisks, including how they affect host cell metabolism, immune responses, and the protein functions, etc. (Designed and drafted in Adobe Illustrator cs5)

The innovative approach used in Zheludev’s group certainly provides a promising path to study undiscovered viroids or other microbial entities. The study reveals the RNA elements in the microbiome that have been "invisible" for us, not only providing important research ideas for the interdisciplinary exploration of microbiome, virology and genomics in the future, but also representing at the potential diversity and complexity of virus-like elements in nature. The discovery of viroid - like colonists in the human microbiome is a relatively new and exciting development in microbiome research. While there is still much to be learned, the available evidence suggests that these entities may have significant implications for human health and disease. For example, Wu et al. discovered CCAV in colorectal cancer tissues and demonstrated through in vitro experiments that its expression may be associated with viral infection, immune dysregulation, and tumorigenesis [3]. However, further research is needed to elucidate the exact mechanisms by which viroid-like elements may function in the human microbiome. Future research should focus on improving detection and identification methods, developing in vitro and animal models for viroid - like elements, and exploring their functional roles in the human microbiome. By addressing these challenges, we can gain a better understanding of the role of viroid-like colonists in the complex ecosystem of the human body and potentially develop new strategies for preventing and treating diseases associated with these enigmatic entities. A more extensive compilation of related works will also be crucial for consolidating the knowledge in this emerging field and guiding future research directions. Additionally, interdisciplinary collaborations between microbiologists, virologists, geneticists, and clinicians will be essential for advancing our understanding of the complex interactions between viroid-like elements and the human host.

## Data Availability

The authors confirm that the data supporting the findings of this study are available within the article.
